# From abortion-inducing medications to Zika Virus Syndrome: 27 years experience of the First Teratogen Information Service in Latin America

**DOI:** 10.1590/1678-4685-GMB-2018-0111

**Published:** 2019-04-11

**Authors:** Lavinia Schüler-Faccini, Maria Teresa Vieira Sanseverino, Alberto Mantovani Abeche, Fernanda Sales Luiz Vianna, Lucas Rosa Fraga, Anastacia Guimaraes Rocha, André Anjos da Silva, Paulo Ricardo Assis de Souza, Artur Hartmann Hilgert, Camila Pocharski Barbosa, Caroline Grasso Kauppinem, Daniela Fernandes Martins, Daniela Silva Santos, Gabriel Henrique Colpes, Gabriela Ecco, Helena Margot Flores Soares da Silva, Louise Piva Penteado, Tatiane dos Santos

**Affiliations:** 1 Sistema Nacional de Informação sobre Agentes Teratogênicos (SIAT), Hospital de Clínicas de Porto Alegre, Universidade Federal do Rio Grande do Sul, Porto Alegre, RS, Brazil

**Keywords:** Teratogens, pregnancy, zika, thalidomide, misoprostol, rubella

## Abstract

In 1990, the first Teratogen Information Service in Brazil (SIAT) was implemented in the Medical Genetics Service at Hospital de Clinicas de Porto Alegre. SIAT is a free-to-use information service both to health professionals and the general population, especially to women who are pregnant or planning pregnancy. The main objective of this paper is to present the activities of SIAT in its initial years (1990-2006), compared to those in the last decade (2007-2017). In addition we review the scientific contribution of SIAT in the field of human teratogenesis. Since 1990, SIAT received 10,533 calls. Use of medications were the main reason for concern, accounting for 74% of all questions, followed by other chemical exposures (occupational, cosmetics, environmental), and maternal infectious diseases. Among its main contributions to scientific knowledge was the collaboration for the identification of two new human teratogens: misoprostol in the 1990s and Zika virus in 2015/16. In conclusion, SIAT is still evolving, as is the Medical Genetics Service that hosts it. Through its 27 years of existence more than 300 undergraduate and graduate students have rotated at SIAT. Presently, SIAT is expanding the research to experimental teratogenesis and to investigation of molecular mechanisms of teratogens.

## Introduction

Teratogen Information Services (TIS) were established after the thalidomide tragedy, and proliferated in North America and Europe with the aim to provide individualized information about embryo-fetal risks associated with maternal exposures during pregnancy. Moreover, these services became important sources of data on teratogenicity studies through the prospective evaluation of the newborns delivered by exposed mothers (Chambers *et al.*, 2011). Although successful in high income countries, they were not able to cover the teratogenic risks for pregnancies in middle and low-income countries like those in Latin America. Here the scenario is characterized by lower educational levels, higher prevalence of infectious diseases, poorer nutrition, lower control for prescription drugs, a culture of self-medication, and sharing of medications among relatives or friends, abortion prohibition, poorer working conditions and, in many instances, exposure to higher levels of environmental pollutants.

Therefore, in 1990, the first Teratogen Information Service in Brazil (SIAT - Sistema Nacional de Informação sobre Agentes Teratogênicos) was introduced in Brazil, based at the Medical Genetics Unit (presently Medical Genetics Service) at Hospital de Clínicas de Porto Alegre, Universidade Federal do Rio Grande do Sul. It was not only the first to be operating in Brazil, but also the first in Latin America, and in a developing country. In 1993, a preliminary report about the SIAT was published by our team in this journal (Schuler *et al.*, 1993), where we described the methodology and the initial results of this program. SIAT was, from its inception to the present day, a free-of-charge telephone based information service, open both to medical doctors and health professionals, and to the general population, especially pregnant women or women planning pregnancy. Newer technologies were added as time went on, and presently SIAT has a webpage, receives questions through e-mail, and replies with written information (statements) to all doctors responsible for the woman who generated the enquiry. Furthermore, SIAT moved from being only a passive question-answering service to a service that acted pro-actively in screening for teratogens in Brazil, and, to a reasonable extent, in other developing countries that had similar common maternal exposures and risk factors to ourselves. SIAT soon integrated with the ENTIS (European Network of Teratogen Information Services), and more recently joined also the OTIS (Organization of Teratogen Information Services, which coordinates the North American TIS).

However, in the almost three decades of operation of SIAT, the economic and health indicators both in Brazil and in the rest of Latin America have changed substantially, with an impressive impact on infant and perinatal mortality. This has decreased by 47% from 1990 to 2007, and under-five mortality due to congenital disorders has become the second leading cause of infant mortality, superseded only by perinatal causes (Victora *et al.*, 2011). This changing scenario could have an impact in the demographics, as well as in the types of exposures of women who contacted the SIAT over the years. Therefore, the main objective of this paper is to compare the demographics of the women who contacted the SIAT in its initial years, including their questions and exposures, with those who contacted us in the last decade. In addition, we will review the scientific contribution of the SIAT to knowledge of human teratogenesis and its future perspectives.

## Subjects and Methods

### Information Service

SIAT’s basic operational system was already described (Schuler *et al.*, 1993). Calls are received by phone, fax, and e-mail. A structured interview is performed by especially trained medical students, where not only the main reason of call is asked, but also other variables are questioned that might constitute additional risk factors: place of residence, profession, work activities, educational level, maternal and paternal age, obstetric history, family history including consanguinity, pre-pregnancy and pregnancy maternal weight, chronic or acute diseases, alcohol intake, smoking habits, and use of other drugs and other exposures, especially use of medications. When the call comes from a medical doctor, or other health professional, a questionnaire is sent through e-mail to be filled out and returned to us. Answers are given verbally to lay callers (pregnant or planning pregnancy women mostly) and a written answer is sent to her doctor if she agrees. Calls received directly from health professionals always receive a written statement. Besides the reasons that generated the call, this written statement includes all other potential risks identified through the structured questionnaire and also general information about prevention of birth defects (e.g., folic acid intake, alcohol intake, and smoking). Finally, a general conclusion is provided where advice is sent to the doctor, with a summary including a risk/benefit balance tailored for each case.

### Information sources and databases

Information for each reason of call, or “exposure”, is reviewed in the scientific literature (PubMed and SciELO), as well as in specific data banks like REPROTOX® (Fitzpatrick, 2008) This information is then summarized in Portuguese and stored in our own SIAT data bank. Every time a new call is received for an exposure previously queried, a new literature review is made, and the summary is updated.

### Research

Specific research projects have been developed by the SIAT team, mainly through the prospective follow-up of pregnancies and through newborn evaluation in cases where maternal exposures had been referred to us. After the predicted date of birth, women are re-contacted by the SIAT team and questioned about the outcome of the pregnancy, and especially about the presence of congenital anomalies in the newborn. Whenever possible, babies are examined by one of our medical geneticists. In those cases where direct examination is not possible, a questionnaire is sent to the pediatrician, and sometimes to another medical geneticist in charge of the baby.

### Team

SIAT’s team is composed of medical and pharmacy students who are responsible for taking the calls, reviewing the literature, and organizing a draft of the statements to be sent to the doctor. Every statement is then reviewed by one of the five coordinators, who are PhDs and experts in different areas of obstetrics, teratology, and genetics.

### Data storage and analysis

Two periods were used for comparison: 1990-2006 and 2007-2017. These periods were chosen intuitively, mostly because of changes in the Brazilian epidemiological profile that was reflected instantly in the reason for calls to SIAT. In 2006, confirmed cases of rubella had officially dropped by 98% in the Americas (Ministério da Saude, 2008), and in 2005, misoprostol was withdrawn from pharmacies due to its over-the-counter and non-approved use as an abortion-inducing agent. Moreover, a website with general information was implemented in 2008, where many common reasons for calls were included, allowing open access for lay people and medical doctors (SIAT, 2017). A Chi-square test was used for comparisons.

### Ethics statement:

This research was approved by the Institutional Review Board from the Hospital de Clínicas de Porto Alegre (69694217.0.0000.5327).

## Results

### Information Service

Table 1 presents a general overview of the main subjects who requested information from SIAT. From August 1990 to December 2017, SIAT received 10,533 calls: 6,503 from 1990 to 2006 (mean 382.5/year) and 4,030 from 2007 to 2017 (mean 366.4/year). Comparing the two periods, there was a decrease in the number of calls about pregnant women, which presently represent almost half of the sample. On the other hand, calls before a planned pregnancy have increased significantly, from only 10% in the early years to 23% in the last 11 years. The profile of the callers has changed as well. Medical doctors were the main callers to SIAT in both periods. However, the proportion of calls from lay people has decreased from 36% (women plus relatives) in the first period to only 25% in the most recent years.

**Table 1:** Profile of index cases and callers in the two different periods.

	1990-2006 (Total N = 6503)	2007-2017 (Total N = 4030)
	N(%)	N(%)
**Index cases** ^a, b^		
Pregnant women	3891 (64.47)	1974 (50.81)
Researchers	685 (11.35)	436 (11.22)
Women planning pregnancy	661 (10.95)	910 (23.42)
Child with congenital defect	517 (8.57)	316 (8.13)
Paternal exposures	165 (2.73)	111 (2.86)
Use of medications during lactation	116 (1.92)	138 (3.55)
**Callers** ^c, d^		
Medical Doctor	3200 (49.21)	2370 (67.39)
Index women	2181 (33.54)	908 (25.82)
Researchers	685 (10.53)	80 (2.27)
Other professionals	267 (4.11)	159 (4.52)
Husband / Relative	170 (2.61)	0 (0.0)

a Number of valid cases: n = 6035 between 1990-2006 and n = 3885 between 2007-2017;

b *p*-value < 0.0001;

c Number of valid cases: n = 6503 between 1990-2006 and n = 3517 between 2007-2017;

d *p*-value < 0.0001.

Although open to calls from any country, SIAT serves essentially as a Brazilian service. Questions from abroad account presently for less than 1% of calls, mostly from countries in Latin America (Table 2). Furthermore, more than one third of the calls came from locations outside the South of Brazil, where we are geographically placed. In both periods, the majority of women were highly educated, but in recent years the proportion of women with university grades has increased from 41% to 69%. Another significant change was in maternal age. Teenage pregnancies have decreased from 5% to a little less than 2%, and pregnancies of women over 35 years old have increased from 23% to 34% (Table 2)

**Table 2:** Demographic characteristics of the index women

	1990-2006	2007-2017
	N (%)	N (%)
**Geographical origin of the call** ^a, b^		
South	3097 (68.07)	1913 (61.12)
Other Regions	1374 (30.20)	1195 (38.18)
Other Countries	79 (1.74)	22 (0.70)
**Scooling** ^c, d^		
Illiterate	17 (0.43)	1 (0.06)
Incomplete Elementary School	236 (5.91)	32 (2.01)
Elementary School	513 (12.85)	58 (3.65)
High School	1556 (38.98)	389 (24.47)
Academic Degree	1670 (41.83)	1110 (69.81)
**Maternal age (years)** ^e, f^		
< 20	262 (5.59)	52 (1.97)
20 - 34	3317 (70.79)	1677 (63.38)
> 35	1107 (23.62)	917 (34.66)

a Number of valid cases: n = 4550 between 1990-2006 and n = 3130 between 2007-2017;

b *p*-value < 0.0001;

c Number of valid cases: n = 3992 between 1990-2006 and n = 1590 between 2007-2017;

d *p*-value < 0.0001;

e Number of valid cases: n = 4686 between 1990-2006 and n = 2646 between 2007-2017;

f *p*-value < 0.0001.

Table 3 shows the main reasons for calls to SIAT. The 10,533 calls included 20,465 questions, representing a mean of around two questions per call. However, the variation was from just one question to 18 different questions in just one patient. The total number of registered exposures was 1,145 (examples of exposures are: aspirin, hyperthermia, rubella, X- Ray, and so on). Use of medications were, in both periods, the main reason of concern, corresponding to almost three fourths of all questions. The relative contribution from other reasons for calls had some small but significant changes, noteworthy maternal infections and vaccinations; these decreased significantly.

**Table 3:** Reasons for Calls (Exposures)^a^.

	1990-2006 (Total N = 12486)	2007-2017 (Total N = 7979)
	N (%)	**N** (%)
Medications	9360 (74.96)	5930 (74.32)
CNS^b, c^	2282 (24.40)	2784 (46.95)
Anti-infective	1231 (13.10)	513 (8.65)
Painkiller	681 (7.30)	233 (3.93)
Inducing-Abortion Substances	394 (4.20)	48 (0.81)
Other substances (Occupational, hair dye, pesticides, etc.)	1367 (10.95)	670 (8.40)
Infections and Vaccines	611 (4.89)	245 (3.07)
Radiation	491 (3.93)	163 (2.04)
Smoking/Alcohol/Recreational Drugs	234 (1.87)	152 (1.91)
Advanced Maternal Age	60 (0.48)	6 (0.08)
Congenital Malformations	58 (0.46)	11 (0.14)
Maternal Diseases (excluding infections)	51 (0.41)	92 (1.15)
Consanguinity	15 (0.12)	3 (0.04)

a *p*-value < 0.0001;

b Main classes of medications;

c *p*-value < 0.0001. CNS: Central nervous system.

When analyzing only the medications, there is a striking difference in exposures between the first and second periods. Enquires about medications acting on the central nervous system were in both periods the most frequent reason for call, but they almost doubled in the recent years (24% vs. 46%). The three other main classes were antibiotics (13% vs. 8%), analgesics (7% vs. 3%), and medications considered to act as abortion agents, which significantly decreased from the first period to the second (4% vs. 0.8%)

### Research

Selected examples of SIAT published research is show in Table 4. Among its main contributions to scientific knowledge was the collaboration in the identification of two new human teratogens: misoprostol in the 1990s and Zika virus in 2015/16. On the other hand, SIAT research provided further evidence for the absence of risk of the rubella vaccine during pregnancy, and the life-saving importance and safety of antiviral treatment of pregnant women with H1N1 flu. The SIAT team also worked on the identification of new cases of the thalidomide syndrome in Brazil, and proposed strategies for epidemiological surveillance of defects compatible with this syndrome. More recently, the SIAT team started to investigate the molecular mechanisms of teratogenesis of thalidomide. In 2001, SIAT published a “Manual of Teratogenesis”, a book with information in Portuguese directed to health professionals (Sanseverino *et al.*, 2001).

**Table 4:** Selected SIAT studies and its contribution to the field of human teratogenesis.

Exposure	Type of study	Main findings	Author, year
**Medications**			
Thalidomide	Case series	Description of three cases of TE occurred in Brazil after 2005.	Schuler-Faccini *et al*., 2007
	Pharmacovigilance	Hospital-based epidemiological surveillance.	Vianna *et al*., 2011
	Case series	Description of two cases of TE in regions with a high prevalence of leprosy and the development of a checklist for recognition of TE phenotypes.	Vianna *et al*., 2013
	Case series	Follow-up of TE cases born between 1959 and 2010.	Kowalski *et al*., 2015
	Pharmacovigilance	Hospital-based epidemiological surveillance.	Vianna *et al*., 2015
	Molecular	Analyses of genetic variants related to TE.	Vianna *et al*., 2013; Vianna *et al*., 2016; Kowalski *et al*., 2015; Kowalski *et al*., 2017;
	Review	History of thalidomide use in Brazil and the occurrence of new cases of TE.	Vianna *et al*., 2017
Misoprostol	Case-control	Misoprostol associated to the occurrence of Moebius sequence in newborns exposed to the medication during pregnancy.	Pastuszak *et al*., 1998
	Case-control	Misoprostol associated to the occurrence of defects of vascular disruption in newborns.	Vargas *et al*., 2000
Venlafaxine	Prospective multicentre cohort	No evidences of increased risk for major malformations.	Einarson *et al*., 2001
Loratadine	Prospective multicentre cohort	No association for increased risk for major malformations.	Moretti *et al*., 2003
**Infections and Vaccines**			
Rubella Vaccine	Prospective cohort	Vaccine safety during pregnancy, presenting no risk for congenital anomalies.	Minussi *et al*., 2008
H1N1 virus and oseltamivir	Prospective cohort	No increased risk for major anomalies in exposures to H1N1 or oseltamivir.	Silva *et al*., 2014
Zika Virus	Case series	Suggestion of a possible association between maternal infection by Zika virus and congenital microcephaly.	Schuler-Faccini *et al*., 2016a
	Critical Review	Discussion and confirmation of Zika virus as a new human teratogen.	Schuler-Faccini *et al*., 2016b
	Case series	Description of 1501 newborns with congenital microcephaly in Brazil.	França *et al*., 2016
	Critical Review	Contribution in the definition of criteria for notification of microcephaly in Brail.	Victora *et al*., 2016
	Case series	Description of the phenotype of Zika virus embryopathy.	Del Campo *et al*., 2017
**Environmental**	Hospital-based	No association for increased risk for major malformations.	Leite *et al*., 2001
	Hospital-based	No association for increased risk for major malformations and other adverse outcomes.	Oliveira *et al*., 2002
**Teratogens in general**	Case-control	Association between maternal alcohol use during pregnancy and criminal behavior	Momino *et al*., 2012
	Review	Evaluation of teratogens in the Brazilian population.	Schüler-Faccini *et al*., 2002; Momino et al., 2003; Garcias and Schuler-Faccini, 2004
	Review and evaluation of congenital anomalies in Brazil	Integrative approach of the principles of teratogenesis, teratogenic mechanisms, and other aspects.	Mazzu-Nascimento *et al*., 2017

## Discussion

The knowledge that environmental agents could harm the unborn baby, particularly after the thalidomide tragedy, led to the need for research and information about other potential teratogens (Chambers *et al.*, 2011). Moreover, not only the risk should be evaluated but also the safety of compounds useful for treating maternal diseases. Therefore, a risk/benefit balance is always of paramount importance in those cases where there is the need to choose the best option both for effective treatment of the mother and preserving the health of the unborn baby at the same time. It should also be pointed out that many calls to SIAT are about non-teratogenic exposures, such as most anti-infective drugs, analgesics, or diagnostic X-Rays, and in these cases maternal reassurance is fundamental to avoid unfounded fears and stress (Chambers *et al.*, 2011). In the absence of leaflets or risk classification tables, a Teratogen Information Service (TIS) provides personalized information that involves a careful risk communication process, offers and alternatives to deal with this risk when detected or unavoidable, for example, the use of anticonvulsants by epileptic women. It should be stressed that the risk interpretation depends on the clinical setting, whether it be, firstly, a woman/couple planning pregnancy, or secondly, a pregnancy where an exposure already occurred, or thirdly where a baby was born with a birth defect and there had been a potential teratogenic exposure (Dathe and Schaefer, 2018).

In Box 1 we present an illustrative case where many daily issues on teratogen counselling are presented and discussed.

In our sample, most of the women were already pregnant when the call to SIAT occurred (Table 1), and in the majority of the cases, the exposure that motivated the call had already occurred. This is mainly due to the fact that not only in Brazil, but all over the world, almost half of pregnancies are not planned, with significant risk of unintentional exposures occurring during the first weeks of gestation (Momino *et al.*, 2003; Han *et al.*, 2005; Hohmann-Marriot, 2017). Medications constituted the major reason for the call in both periods, and this is the same for calls in all TISs around the world. The percentage of around 70% of calls arising from use of medications in SIAT is quite comparable to those in developed countries: 82% in Italy (De Santis *et al.*, 2008); 68.5% in Utah, USA (Campbell *et al.*, 2016); 60% in the USA in general (Hancock *et al.*, 2009). Perhaps the most striking observation from our study is the prevalence of calls about drugs acting on the CNS, which almost doubled in the recent years (from 24% to 46%). CNS prescription drugs are also the commonest class of drugs in different TIS around the world. Kennedy *et al.* (2016) made a review of calls to the Australian/New Zealand TIS comparing two periods (2000-2005 vs. 2006-2011), and they reported a doubling in calls about psychotropic drugs. Gendron *et al.* (2009) in Montreal also reported a majority of calls concerning antidepressants (17%) and benzodiazepines (5.3%). CNS drugs represented 25% of calls to a German TIS (Dathe and Schaefer, 2018). Depression, bipolar disorder and epilepsy are prevalent among women in reproductive age, and this fact might explain the high prevalence of calls on CNS prescription drugs.

On the other hand, abortion-inducing medications and maternal infections are the peculiar situations of a TIS in a country like Brazil, and possibly other low and middle-income countries. The legal prohibition of abortion in Brazil is reflected in the expressive number of calls from women that tried unsuccessfully to get an abortion by her own ways. At the beginning of the 1990s, misoprostol, a prostaglandin E compound, freely available as an over-the-counter medication, was commonly used by these women (Costa, 1998). SIAT performed an effective screen to detect the spreading use of misoprostol (4% of calls about medications in the first period), and through the evaluation of the newborns, showed its teratogenic potential when the pregnancy was not lost (Schuler *et al.*, 1992, 1999; Schuler-Faccini *et al.*, 1997; Pastuszak *et al.*, 1998; Vargas *et al.*, 2000). Misoprostol was withdrawn from pharmacies in 2005, and this was probably the reason for the significant drop in calls about abortive medications (4% in the first period vs. 1% in the second)

**Box 1 - Illustrative Case**

Case: A 39-year-old woman, planning her first pregnancy, calls to SIAT after a request from her doctor because she uses carbamazepine and lithium for the treatment of bipolar disorder. She is concerned about the risks of the intake of these medications to her baby. She has already stopped the use of the medications before, but she had severe decompensation and needed psychiatric hospitalization. In the questionnaire carried out by telephone, no other risks were identified for this gestation and she denies other exposures.

Comment: In this case, it is important to note that there is an important disease as an independent risk factor for gestation, mother and baby; therefore, the maternal condition needs to be treated. Both medications offer risks for the development of the baby. Carbamazepine is known to increase the risk for neural tube defects, and the use of lithium during pregnancy has been associated with an increased risk for cardiac malformations (Ebstein anomaly), but much less than initially proposed in the literature. At the same time, these drugs have been efficient in the control of the bipolar disorder in this woman, and in this case, it would be justified for them to be continued throughout her pregnancy. In any case, monotherapy should be the goal, when possible. The dose of both medications has to be checked on a regular basis, which should be as low as possible to reach the treatment. The use of folic acid must be prescribed in doses higher than usual. Follow-up ultrasonography scans should be offered, especially after lithium exposure in the first trimester; a detailed fetal echocardiogram can also be performed in order to evaluate the heart development. It is essential that the woman be aware about the risks. The impact of these medications in the pregnancy are always evolving, therefore, the suggestion is to look for Teratogen Information Services. Finally, this woman is 39 years-old, and her age is a risk factor for chromosomal anomalies, and she should be advised about this risk.

Infections are another interesting example of the importance of a TIS in a developing country. During the first period of our analysis, suspicion of rubella infection was the reason of calls from 112 women (2%), compared to only eight (0,2%) in the second period, a reflection of the effect of massive vaccination campaigns at the beginning of the 2000s. On the opposite side, Zika Virus was a non-existent threat before 2015. Again, SIAT proved to be sensitive of common exposures in our population. The identification of Zika Virus as a teratogen started from calls from medical geneticists from Brazilian states in the Northeast, asking about microcephaly in babies born to mothers with signs of Zika fever (Schuler-Faccini *et al.*, 2016a,b).

Although concerned mostly with the teratogens prevalent in developing countries, SIAT, as an information service, has itself some biases, especially concerning the profile of the women who seek information. Despite an increase in the degree of education and in maternal age in the general population over the last three decades, in our sample, the levels of academic achievement and advanced maternal age (> 35 years) are much higher when compared to the general population. The illiteracy rate has dropped from 13.6% in 2000 to 7.2% in 2016, whereas the rate of Brazilians having an academic degree rose from 6.8% in 2000 to 15.3% in 2016 (IBGE, 2017). In comparison, illiteracy was associated with only 0.43% of the calls in the first period of SIAT, dropping to 0.06% in the second, and women with an academic degree accounted for 41% of the SIAT sample in the first period and for 69% in the second one. A trend is also observed in the number of women having babies after 35 years of age in Brazil: 8% in 1994, 9% in 2000 and 10% in 2009 (Ministério da Saúde, 2017). Comparatively, maternal age over 35 years was 23% in the first period of SIAT and 34% in the second. This difference between our sample and the general population is believed to be due to the fact that most of the calls came from private clinics rather than public health facilities. Alternatives to reach the under-served population and public health facilities should be one of our goals in the coming years.

In conclusion, SIAT is an evolving program effectively facing challenges to provide medical support for the Brazilian population, as is the Medical Genetics Service that hosts it, and whose 35 years we are commemorating in this special issue. Nevertheless, SIAT has been shown - as have other TIS around the world - to be a sensitive screen of maternal exposures during pregnancy and a powerful instrument to prove teratogenicity or safety, and can be a useful resource to establish policies for prevention of congenital anomalies.

Through its 27 years of existence, more than 200 undergraduate students have rotated at SIAT as part of their medical education, as well as graduate students from different backgrounds (Table S1). Some of them stayed as professionals, expanding the research to experimental teratogenesis and to investigation of molecular mechanisms of teratogens and their interaction with developmental gene networks.

## Supplementary material

The following online material is available for this article:

Table S1 Participants of SIAT since its beginnings.

*Associate Editor: Roberto Giugliani*
