# Supplementary material for: From abortion-inducing medications to Zika Virus Syndrome: 27 years experience of the First Teratogen Information Service in Latin America
Source: Genet Mol Biol. 2019 Apr 11;42(1 Suppl 1):297–304. doi: 10.1590/1678-4685-GMB-2018-0111 (PMC6687353; doi:10.1590/1678-4685-GMB-2018-0111)
Supplement: Supplementary file 1 [file 1415-4757-GMB-1678-4685-GMB-2018-0111-20190327-suppl1.pdf]

Suplementary material to "From abortion-inducing medications to Zika Virus Syndrome: 27 years experience of the First Teratogen Information Service in Latin America"

Table S1 - Participants of SIAT from its beginnings.

Alberto Mantovani Abeche	Douglas Elias C dos Santos	Livia Zart Bonilha
Alice Castro Menezes Xavier	Douglas Marinho De Matos	Louise Piva Penteado
Aline Kives Berger	Eduardo De Araujo Silva	Lucas Rosa Fraga
Aline Mallmann Couto	Eduardo Dytz Almeida	Luciana Dutra Martinelli
Amanda Senna P dos Santos	Eliana Silva Machado	Luciana Johann
Ana Caroline Silveira De Farias	Elisa Cordeiro Apolinário	Luciana Tovo
Ana Cláudia Magnus Martins	Elisa Ruiz Fülber	Luis Fernando Bica Borges
Ana Cristina Bittelbrum	Erica Tatto	Luísa De Jesus Martins
Ana Paula Astarita Sangoi	Estevão Naoto O Gutierrez	Luisa Grave Gross
Anastácia Guimarães Rocha	Fabiana Costa Menezes	Luise Teixeira Poitevin
Andre Anjos Da Silva	Fabiana R Vasquez	Luiza Barboza De Souza
Andre Junior Nicola	Fabiola Doff Sotta Souza	Luiza Metzdorf
Andressa Cardoso De Azeredo	Fabricio Silva Da Costa	Magali Tavares Barata Camillo
Angelica Salatino De Oliveira	Felipe Canello Pires	Marcela Metzdorf
Angélica Sauthier	Fernanda Duarte Torres	Marcelo Bremm
Anne Orgler Sordi	Fernanda Fischer	Marcio Perin
Arthur Ludwig Paim	Fernanda Mezzomo Collares	Marcio Schneider Medeiros
Artur Hartmann Hilgert	Fernanda Sales Luiz Vianna	Maria Aparecida A Leopoldino
Aruza Rodrigues Quintana	Fernando Pecis	Maria Luiza Sanchotene
Barbara Berzan	Fernando Procianoy	Maria Teresa Vieira Sanseverino
Betina Piccoli Franciosi	Flávia Vieira Lopes	Mariana Alves Fonseca
Betina Rodrigues Oliveira	Franciele Darsie Dahmer	Mariana Costa Hoffmeister
Betina Stifelman Katz	Franciele Sabadin Bertol	Mariana Hollmann Scheffler

Bianca Chassot Benincasa
Bianca Gubiani Ferreira
Breno Cordova Matte
Bruna Paulsen Panato
Bruno Florentino Goldani
Bruno Ribeiro Bossardi
Camila Giugliani
Camila Janke Lopes
Camila Pocharski Barbosa
Candice E S Dos Santos
Carlos Eduardo Bastiani
Carmem Vinhas Santos
Carolina Casanova Meneghetti
Carolina Casara
Carolina de Vasconcellos
Carolina Fischinger M de Souza
Carolina Friedrich
Carolina Meira Moser
Carolina Pedone Valdez
Carolina Ribas Do Nascimento
Carolina S M Leite
Carolina Waldmann
Caroline Flores De Oliveira
Caroline Grasso Kauppinem
Caroline Vieira Dos Santos Witt
Caroline Vieira Pinheiro
Caroline Walker

Gabriel Henrique Colpes
Gabriela Camargo
Gabriela Ecco
Gabriela Gayer Scheibler
Gabriela Jacques Hoss
Gabriela Kroeff Schmitz
Gabriela Rossi
Georgea Malfatti
Giordani Rodrigues Dos Passos
Giovanna Sorgato Tessmann
Greyce Berton
Guilherme Pozueco Zaffari
Guillermo Manozzo Trevisol
Gustavo Hirata Dellavia
Haley Calcagnotto Dos Santos
Halim Roberto Bajotto
Helena Margot Flores S Silva
Hernando Augusto Clavijo
Isadora Gubert Zanross
Izabela Rodrigues Ávila
Jamily Pertile
Jane Cronst
Jane Mattei
Joanine Andrighetti Sotilli
João Leonardo F Pietrobeli
Joao Valter Pires Junior
Juliana De Azambuja

Mariana Rangel Ribeiro
Mariela Larrandaburu
Mario Tregnago Barcellos
Marta Haas Costa
Mateus Curbeti Becker
Mauricio Fontoura Ferrao
Michele Morales Dos Santos
Micheli Rita Galvan
Natalia Antulino Fernandes
Nicóli Bertuol Xavier
Nina Stein
Nivio Moreiria
Osvaldo Pinto Artigalas
Patricia Ashton Prolla
Patricia Zanotelli Cagliari
Paula Baptista Sanseverino
Paula Pitta Pinheiro
Paulo Ricardo Assis De Souza
Paulo Vitor Crestani
Pedro Soibelmann Tetelbom
Perla Drescher
Pietro Baptista De Azevedo
Priscila Bellaver
Priscilla Granja Machado
Regina Maria Boaz
Renan Desimon Cabral
Renata Faerman

Cássio Mallmann
Catiele Antunes
Celia Nickel
Cezar Henrique K Vargas
Chádia Lucca El Hajjar
Charles Andre Carvalho
Clarissa Moreira Borba
Clarisse Luisa Stefani
Cristina Beatris Bergamaschi
Cristina Brinckmann O Netto
Cristina Leite
Cristina Rojas Kath
Cristine Sortica Da Costa
Daiana Eltz Martins
Daniel F Gomes Soares
Daniel Rodrigues Conill Gomes
Daniel Tornaim Spritzer
Daniela Fernandes Martins
Daniela Manzke
Daniela Silva Santos
Daniele Sparemberger Oliveira
Denise Gomes
Diego Fraga Pereira
Juliana M Szymanski Finkelsztejn
Juliana Santos Varela
Juliana Starck Wartchow
Juliano Peruzzo
Karen Boianovsky
Karina Carvalho Donis
Karlo Biolo
Katherine Krieser
Kelli Wagner Gomes
Lara Mombelli
Larissa Valency Eneas
Laura Bertoldi Porcello
Laura Moschetti
Lavinia Schüler Faccini
Leandra Rech
Leandro Bombassaro
Leonardo Santos Hoff
Leovegildo Pablo Tondello Martins
Leticia Maria Vaz Dos Santos
Leticia Reisderfer
Leticia Rossetto Daudt
Lígia M Rocha de Azevedo
Livia Andreoni
Rita de Cassia S Azambuja
Roberta Allgayer De Moraes
Rosana Gomes Monteggia
Rossana Mizunski Peres
Simone Mattioti
Simone Oliveira Medeiros
Suzan Brancher Brandao
Taisa B Lopes
Tatiana Ckless Moresco
Tatiane Dos Santos
Tatiane Emmanuele Da Rosa
Tiago Bortolini
Tiago Lansini
Tito Emílio Vanelli Costa
Ursula Maldaner
Vee Wong
Victoria Campos Dornelles
Victória D'azevedo Silveira
Vitor Boschi
Vivane Campesatto
